# Lower incidence of fracture after IV bisphosphonates in girls with Rett syndrome and severe bone fragility

**DOI:** 10.1371/journal.pone.0186941

**Published:** 2017-10-26

**Authors:** Anne-Sophie Lambert, Anya Rothenbuhler, Perrine Charles, Sylvie Brailly-Tabard, Séverine Trabado, Elisabeth Célestin, Emmanuel Durand, Isabelle Fontaine, Lotfi Miladi, Philippe Wicart, Nadia Bahi-Buisson, Agnès Linglart

**Affiliations:** 1 APHP, Department of pediatric endocrinology and diabetology for children, Bicêtre Paris-Sud, Le Kremlin Bicêtre, France; 2 APHP, Reference center for rare disorders of calcium and phosphate metabolism, filière OSCAR and Plateforme d’Expertise Maladies Rares Paris-Sud, Bicêtre Paris-Sud, Le Kremlin Bicêtre, France; 3 APHP, Department of Genetics, Reference Centre for Intellectual Disabilities, Pitié-Salpêtrière, Paris, France; 4 APHP, Department of Genetics and Hormonology, Bicêtre Paris-Sud, Le Kremlin Bicêtre, France; 5 APHP, Rett Center, Necker-Enfants Malades, Paris, France; 6 APHP, IR4M Unit, CNRS-Université Paris-Saclay, Orsay, France; 7 IMP Marie-Auxiliatrice, Draveil, France; 8 APHP, Department of Pediatric Orthopedics, Necker-Enfants Malades, School of Medicine, University Paris Descartes Sorbonne Paris Cité, Paris, France; 9 APHP, Reference center for rare disorders of calcium and phosphate metabolism, filière OSCAR, Necker-Enfants Malades, Paris, France; 10 APHP, Department of Pediatric Neurology, Necker-Enfants Malades, Paris, France; 11 INSERM UMR-1163, Laboratory of Embryology and Genetics of Congenital Malformations, Imagine Institute, Paris Descartes–Sorbonne Paris Cité University, Paris, France; Universita degli Studi dell'Insubria, ITALY

## Abstract

**Background:**

Classic Rett Syndrome (RS) is a disabling condition mainly caused by *MECP2* mutations. Girls with RS are at risk of developing bone fragility and fractures at a young age which results in pain and may seriously impair quality of life.

**Objective:**

To retrospectively assess the safety and efficacy of IV bisphosphonates on fracture, bone mineral density (BMD) and bone markers in RS girls with bone fragility.

**Methods:**

RS girls received either IV pamidronate (n = 19) or IV zoledronate (n = 1) for 2 years.

**Results:**

Of 20 patients studied (age: 12.5 years [6; 39]), 14 were non-ambulatory. The incidence of fracture decreased from 37 fractures in 20 patients, to 1 fracture during or after treatment (follow-up: 3.1 years [1.5; 5]). The spine BMD Z-score improved from -3.2 [-5.6; -0.1] to -2.2 [-3.8; 0.0], p = 0.0006. Most parents reported decreases in chronic pain and 2 patients started to walk. Urinary calcium excretion decreased from 0.7 [0.18; 1.5] to 0.2 [0.03; 0.67] mM/mM of creatinine (p = 0.0001). Pamidronate was well tolerated.

**Conclusion:**

RS girls should be screened for impaired bone mineralization and preventive measures should be taken. In girls experiencing fractures, IV bisphosphonates constitute a beneficial adjuvant treatment to diminish the risk of fracture and restore bone density.

## Introduction

Classic Rett syndrome (RS), although considered rare, is one of the most common causes of intellectual disability in females with an incidence of 1 in 10 000 by the age of 15 years [1]. Most individuals with RS bear a heterozygous mutation in the *MECP2* gene [2]. Clinical outcomes for this syndrome are complex, with varying degrees of autonomic dysfunction, motor impairments influencing mobility, epilepsy, nutritional problems and poor growth [3] [4]. The natural evolution of disease tends towards a loss of mobility during adolescence [5] It has been demonstrated previously that total and spine bone mineral density (BMD), bone mineral content, and bone volume are significantly reduced in children with RS [6] [7]. In addition, RS patients have a high risk of scoliosis and fractures from minimal trauma [8] [9].

**Fig 1:**
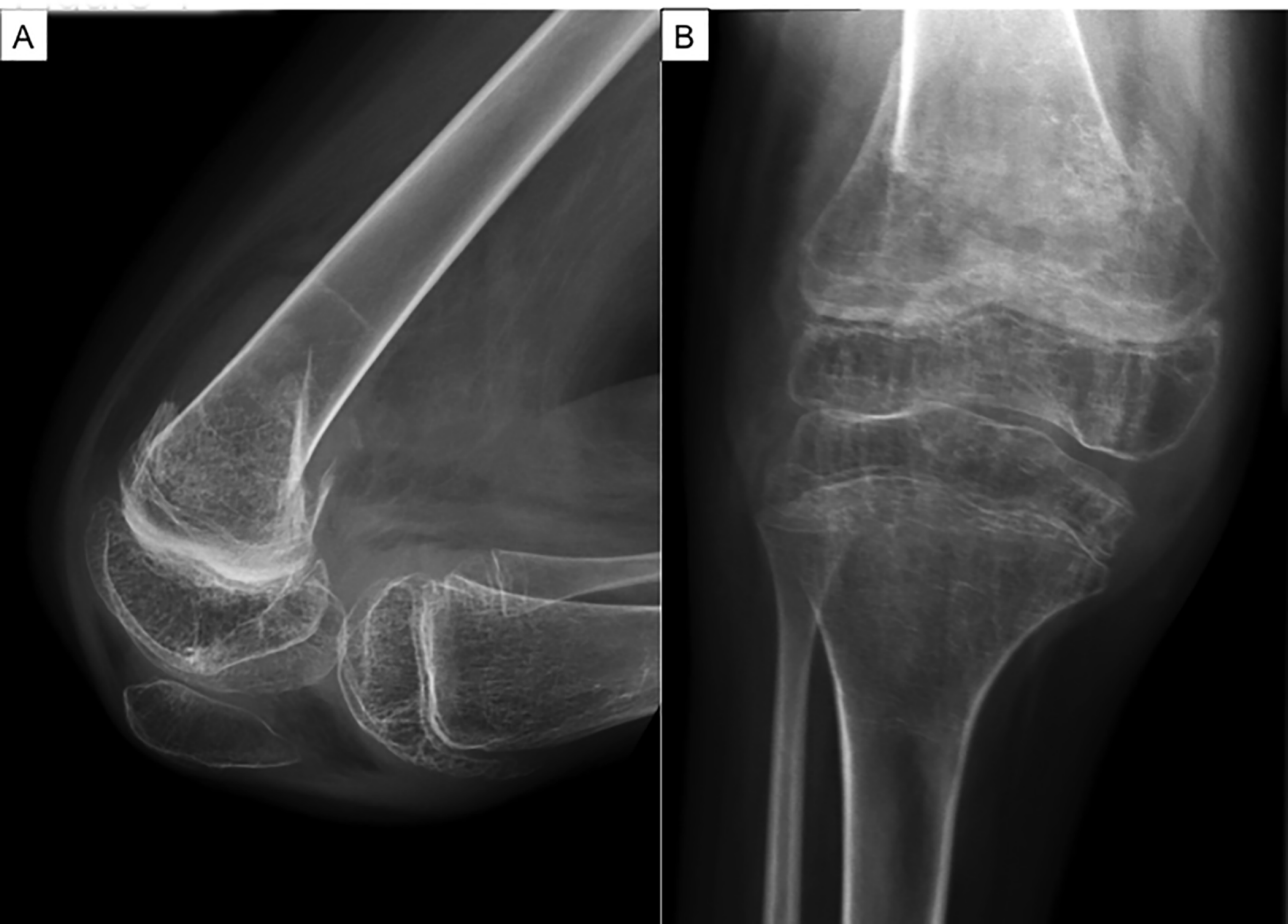
Supracondylar fracture in a RS patient. A: Lateral; B: Face. Typical osteoporotic bone (thin cortices and reduced trabecular bone mass).

Bone fragility in children is characterized by low BMD and a history of fracture [10]. In girls with RS, bone fragility can result in pain and seriously impair their mobility and quality of life [11] [12]. In series reported in the literature, the fracture rate in RS is nearly four times that in the general population. Moreover, many patients present with more than one fracture [8] [13]. The fractures occur predominantly in the long bones of the upper and lower limbs [8] and are significantly more prevalent in non-ambulatory patients [12] [13] (and personal data). Fractures often require surgical intervention for optimal outcome and are associated with significant morbidity, related to malunion or non-union, all of which can cause lifelong disability (**Fig 1**).

The use of bisphosphonates to treat bone fragility has been rigorously evaluated in large-scale clinical trials in adults [14]. Pamidronate, a nitrogen-containing bisphosphonate, is the standard treatment for children with moderate-to-severe osteogenesis imperfecta [15] and was found to be a safe and effective agent to increase BMD in non-ambulatory children with cerebral palsy [16]. Only one case report has described the impact of pamidronate in a girl with RS. In this patient, the BMD z-score increased dramatically upon treatment (-3.8 to -1.3), and she did not experience any further fractures after starting therapy [17]. Our aim was to assess the safety and efficacy of bisphosphonates to treat bone fragility in a cohort of RS patients who presented with fractures and/or bone pain.

## Methods

### Study design

We analyzed the health records of 20 patients affected with classic RS and confirmed mutations in *MECP2* (**S1 Table**) who received pamidronate therapy in our center between January 2009 and December 2016. Patients were put on IV bisphosphonates therapy for bone fragility (i.e., z-score of BMD ≤ -2 and at least one fracture) or bone pain and low BMD (i.e., z-score of BMD ≤ -2).

### Treatment protocol

Pamidronate (3-amino-1-hydroxypropylidene-bisphosphonate) was administered once a day on two consecutive days, and this was repeated at 3-month intervals for 2 years. Each daily dose was 0.75 mg pamidronate/kg of body weight, administered intravenously over 4 hours in a volume of 200 mL. One patient received IV zoledronate at a dose of 0.04 mg/kg every 6 months for 2 years because she could not be hospitalized for several days.

### Calcium and vitamin D supplementation

Daily calcium intake was quantified in all patients by a dietician. Patients with low calcium food intake received a daily calcium supplement ranging from 500 to 1000 mg/day with the aim of reaching the recommended intake for age. The daily dose of calcium supplements was increased to 1000 mg/day for 7 days before, during, and for 7 days after the bisphosphonate administration. Additional vitamin D supplementation (100,000 IU) was given routinely every 3 months to all 20 patients.

### Measurements

The following data were recorded at each hospital visit: body weight (kg and SD), height (cm and SD) and body mass index (BMI) (kg/m^2^ and z-score), Tanner stage, mobility measured through the Gross Motor Function Classification System (GMFCS), daily caloric intake, number of fractures and injuries, total calcium, serum phosphate, alkaline phosphatase, serum creatinine, circulating 25OH vitamin D (25OHD), and serum PTH levels, urinary calcium to creatinine ratio (noting that 24-hour urine collection is not feasible in patients with cerebral palsy), and all prescribed medications, particularly any that might influence bone mineralization.

BMD was measured by dual-energy x-ray absorptiometry (DXA). Measurements were taken prior to the pamidronate therapy, at the end of the 2-years of pamidronate therapy, and approximately once a year thereafter. Whenever possible, BMD measurements were taken at the lumbar spine. Because of previous spine surgery or extremely severe scoliosis, for some patients BMD was measured and recorded at the femoral neck but not included in the data analysis. Pain was not prospectively assessed or recorded. When comments about pain made by parents were noted by the physicians in medical files, they were recorded.

### Statistical analyses

Differences between time points were assessed using paired nonparametric tests (Wilcoxon test). The significance threshold was set at p ≤ 0.05. Statistical analyses were carried out using GraphPad PRISM (v6). All values are presented as median (minimum, maximum).

### Participants consent

The research is retrospective and analyses the current practices in a cohort of patients affected by Rett syndrome. According to the Jardé law in France, the study was approved by the French National Data Processing and Liberties Commission (CNIL). The need for written consent is therefore waived by this law. Patients were informed orally of the content of the study and their consent obtained. They have the right claim their opposition (i.e. refuse to participate) to the study at any time by sending a correspondence to http://recherche.aphp.fr/eds/droit-opposition.

## Results

### Patients

A total of 20 patients with RS and bone fragility were included in our retrospective analysis (**Table 1**). Median ages at diagnosis of RS, start of therapy and at last follow-up assessment were 3.0 years [0.9; 39.0], 12.5 years [6.0; 39.0], and 14.3 years [8; 43], respectively.

**Table 1:** Characteristics of the 20 patients at the start of bisphosphonate therapy.

	Median	Min-Max	Normal range
Age at diagnosis (years); n = 20	3.0	0.9; 39.0	NA
Age at start of therapy (years); n = 20	12.5	6.0; 39.0	NA
Age at last assessment (years); n = 20	14.3	8; 43.0	NA
Body mass index* (kg/m^2^/z-score)	13.9 / -1.5	11; 19.04 / -2.4; 0.8	NA/ -2.0 to 2.0
Tanner stage**	B1, n = 8; B2, n = 1; B3, n = 3; B4: n = 1; B5, n = 7	NA	NA
Caloric intake (kcal/day; % of the recommended intake for age)	1500; 87%	960; 2500	NA
Food calcium intake (% of recommended intake for age)	756; 83%	560–1300	NA
Nutritional support (enteral nutrition through gastrostomy)	n = 4	NA	NA
Ambulation (yes/no)	5/15	NA	NA
GMFCS level	5 (III); 15 (V);	I-V	NA
Antiepileptic therapy	18 patients		
25OH vitamin D (ng/mL)	35	17; 76	20 to 60
Urinary calcium/creatinine ratio (mM/mM)	0.7	0.18; 1.5	0.1 to 0.5
Urinary deoxypyridinoline /creatinine ratio (mM/mM)	18.4	6.6; 34.8	10 to 40 (depending on age)
Serum crosslaps (ng/mL)	1.1	0.2; 2.3	0.2 to 3.5 (depending on age)
Alkaline phosphatase (IU/L)	125	57; 296	100 to 550 (depending on age)
Osteocalcin (ng/mL)	41	13; 91	49 to 118

NA: not appropriate

* in this table we report the values at the start of pamidronate therapy

**B for breast development, the Tanner stage ranges from B1 (no signs of breast development) to B5 (complete pubertal maturation of the breasts)

The median BMI was initially 13.9 kg/m^2^ [11; 19.04] (median z-score -1.5 [-2.4; 0.8]). Amongst the 16 patients for which we were able to calculate the BMI, thirteen were underweight, as indicated by a BMI below 17 kg/m^2^ in women (patients older than 18 years) or a BMI z-score below -2 in girls (patients younger than 18 years). Out of the 20 patients, 13 had insufficient daily caloric intake and 15 had insufficient calcium food intake, while 4 already received enteral nutritional support and 14 received regular 25OHD supplementation prior to the bisphosphonate program. The median 25OHD level was 35 ng/mL [17; 76].

Regarding medication, 18/20 patients were on anticonvulsant therapy, including sodium valproate in 12/18 cases. Out of the 14 patients older than 10 years at the start of therapy, 3 had primary amenorrhea and 4 patients had a pubertal delay. Five girls were able to walk at least three steps without support and considered ambulatory, being classified as GMFCS level III, unlike the others who were classified as GMFCS level V (limited in their ability to maintain antigravity head and trunk postures and control leg and arm movements). We did not quantitatively assess pain before or during the therapy through a standardized scaled pain system. We did, however, find evidence in the health records that 16/20 parents mentioned chronic pain and/or discomfort during manipulations before the initiation of therapy.

Nineteen of the patients received pamidronate IV, with a median total dose of 9.4 mg/kg [8.2; 12]. The other patient was treated with zoledronate at a dose of 0.16 mg/kg.

### Fractures

**Fig 2:**
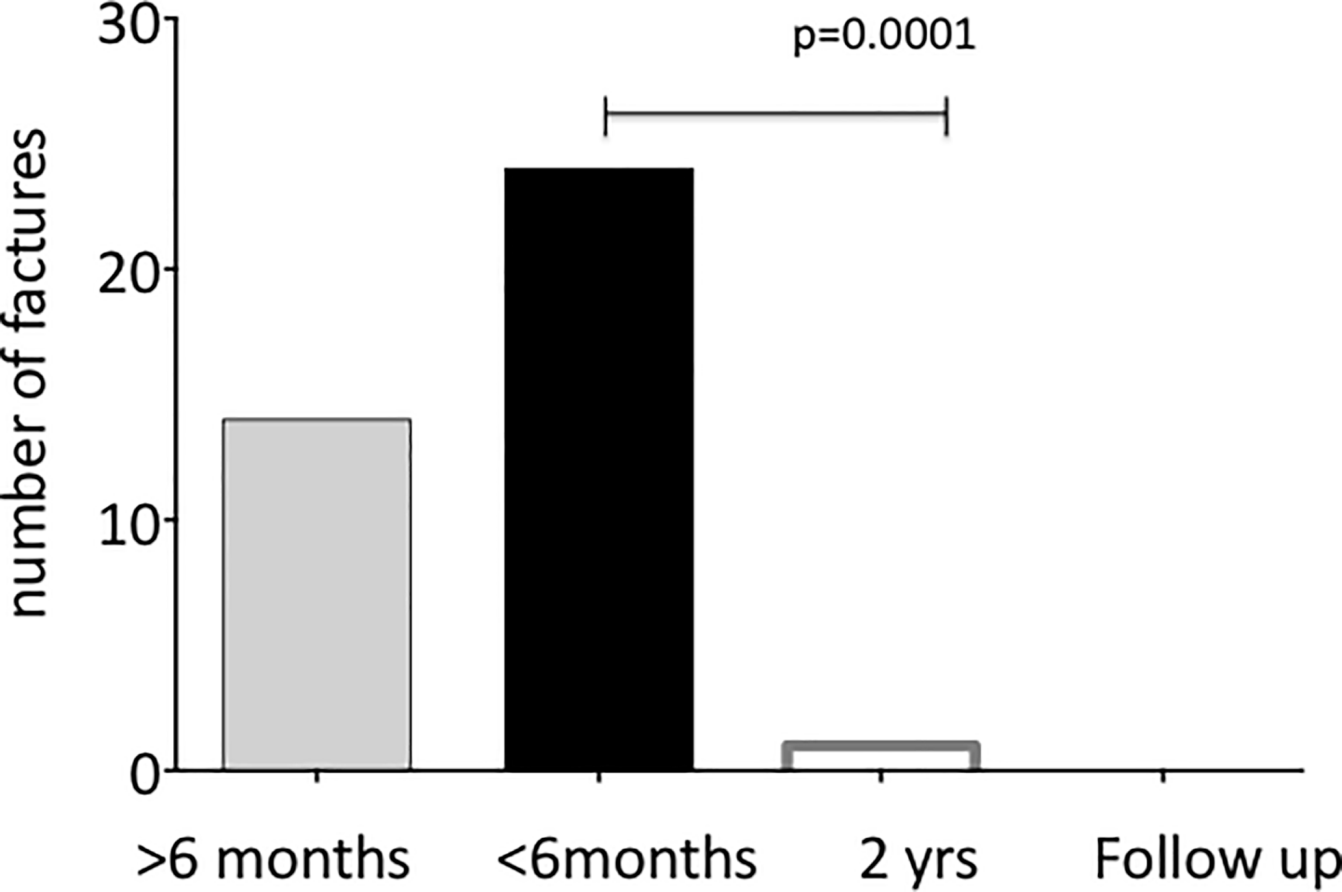
Fractures before, during and after bisphosphonates. Number of fractures reported by the parents/guardians or diagnosed on vertebral X-rays before bisphosphonate therapy (grey and black bars), at the end of therapy (white bar) and at follow-up (no bar).

Overall, 13/20 patients had experienced at least one fracture before the treatment. In total, we identified 37 fractures (24 in the 6 months prior to the start of therapy) (**Table 2**), which represents a median of 2 fractures per patient (range 0 to 8). Fractures were diagnosed as an unexpected finding in 3/13 patients and after minor trauma in 10/13 patients, occurring together with altered mobility in all cases. Most affected the long bones. Only one patient had a new fracture during the two years of bisphosphonate therapy, and the total length of follow up for this patient was 2.2 years (**Fig 2**). The median length of follow-up was 3.1 years [1.5; .5].

**Table 2:** Sites of the 37 fractures that occurred in the period preceding the bisphosphonate therapy.

Fracture site	Number of patients
Femur	5
Tibia	6
Humerus	5
Foot	4
Spine	4
Not documented	13

### Bone mass density

**Fig 3:**
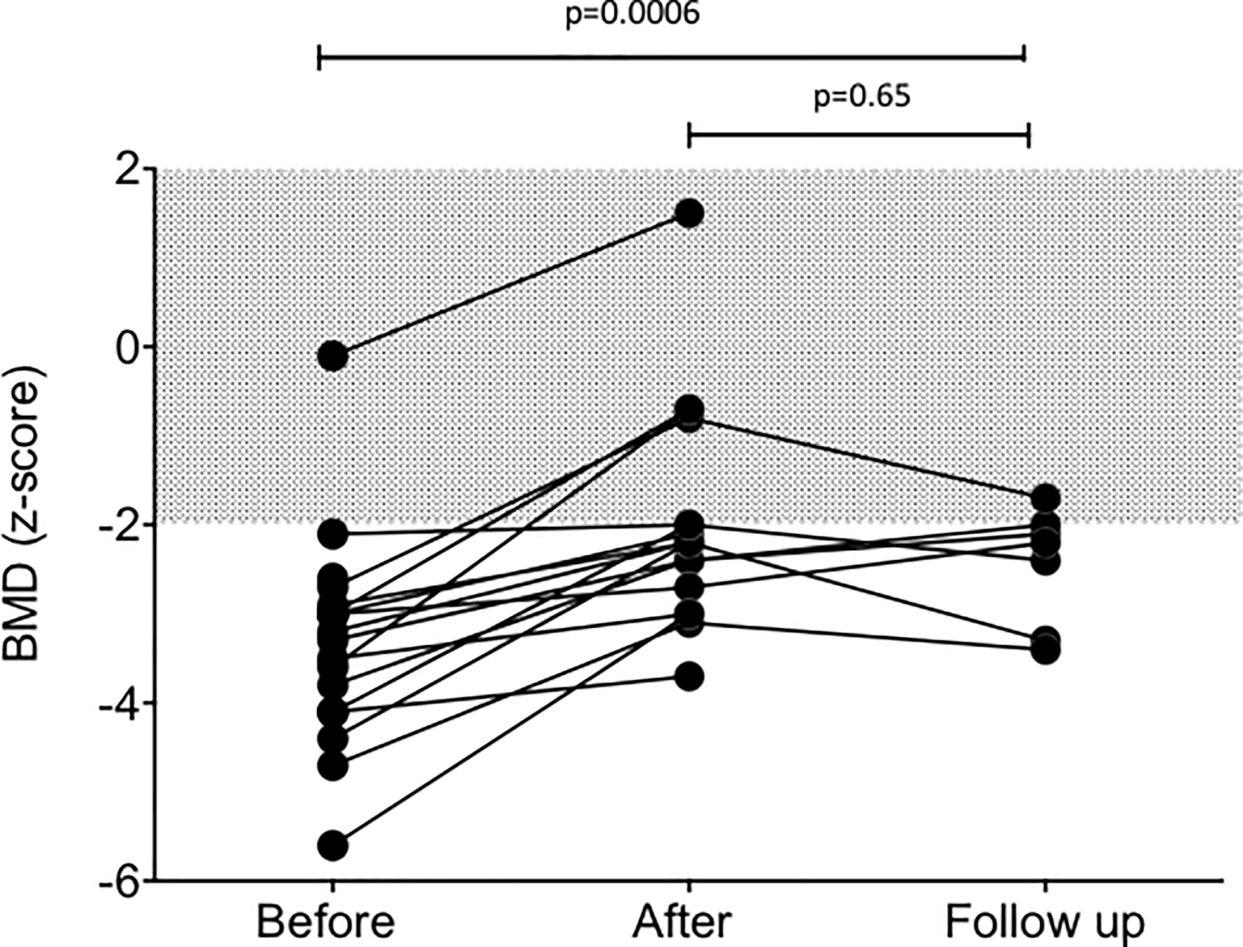
Evolution of BMD. Changes in BMD at the lumbar spine in 18 RS patients from baseline to the end of treatment (after) and up to the last post-treatment visit (follow-up). Data are not shown for two patients who had spine arthrodesis preventing DXA at the vertebrae. Note that one patient has a normal BMD at start of therapy (z-score: -0.1); she had low BMD z-scores at the femoral necks (-2). The shaded area indicates the normal range.

During 2 years on bisphosphonates, the median spine Dual X-ray Absorptiometry (DXA) BMD Z-score improved progressively from -3.2 [-5.6; -0.1] to -2.2 [-3.8; 0] (p = 0.0006). Moreover, it remained stable during follow-up, without ongoing therapy (at 3.1 years [1.5; 5], DXA BMD Z-score is -2.2 [-3.4; -1,7], p = 0.65 [n = 7]) (**Fig 3**).

### Biochemical markers of calcium metabolism

**Fig 4:**
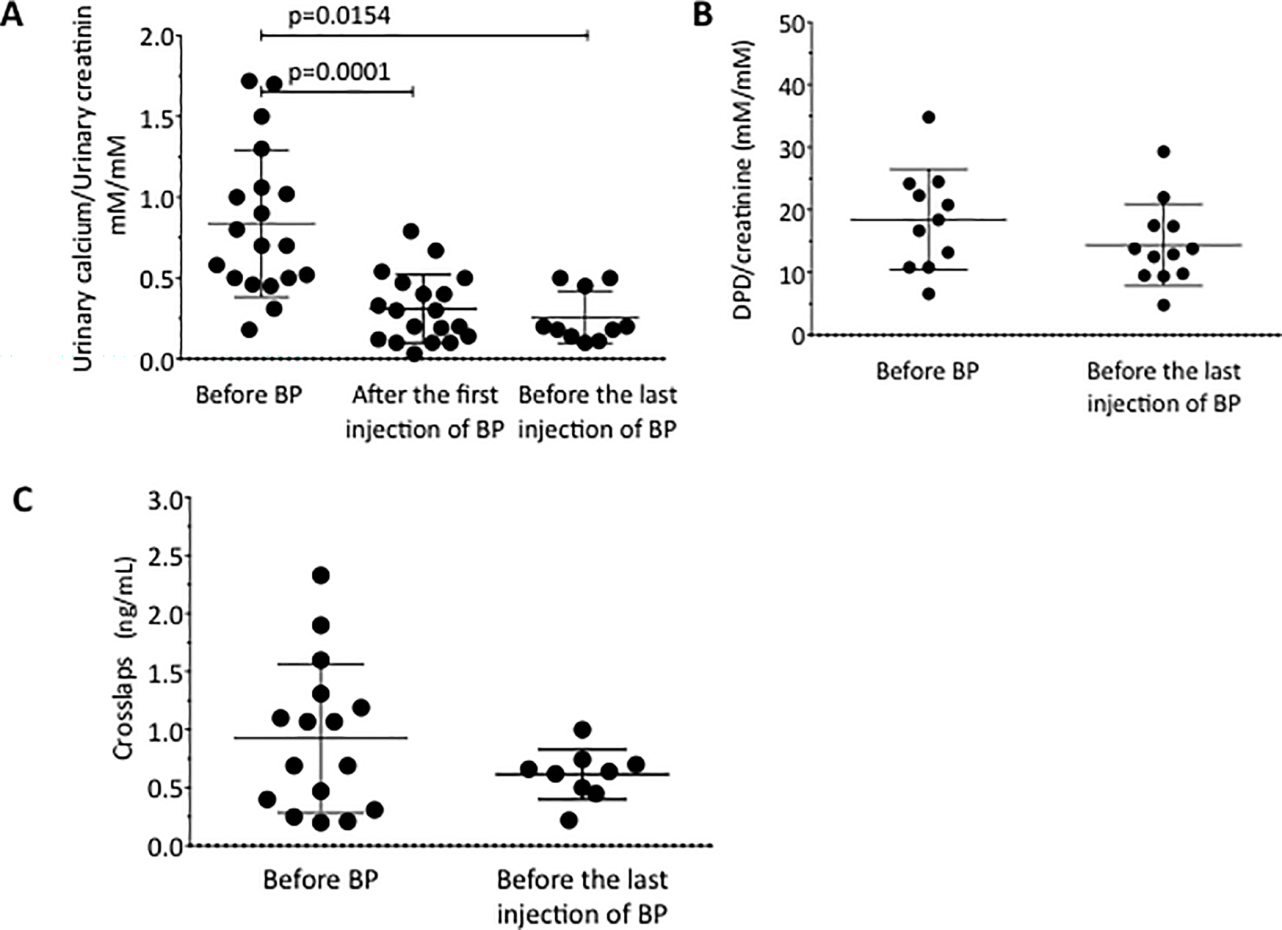
**Changes in the biochemical markers of bone resorption over the course of treatment** (panel **A**: urinary calcium; panel **B**: deoxypyridinoline; panel **C**: Crosslaps).

The urinary excretion of calcium, which likely reflects bone resorption in this context of low calcium intake and lack of ambulation [18] was above the normal range at the initiation of therapy in 14/20 patients (**Table 1**). After the first infusion of bisphosphonates, the urinary calcium to creatinine ratio decreased significantly from 0.7 [0.2; 1.5] to 0.2 [0.0; 0.7] mM/mM of creatinine (p = 0.0001). Overtime, the urinary excretion of calcium decreased progressively reaching a value of 0.2 [0.1; 0.5] mM/mM of creatinine at the end of therapy, significantly lower than before the start of bisphosphonates (p = 0.0154) (**Fig 4A**), and within the normal range. The urinary excretion of the deoxypyridinoline and the serum level of crosslaps, reflecting bone resorption, did not change significantly over the 2 years of bisphosphonate therapy (**Fig 4B and 4C**). Notably, alkaline phosphatase and osteocalcin, markers of bone formation, were at the lower range of normal at initiation of treatment and remained low throughout the treatment period. Alkaline phosphatase and osteocalcin values were not different between pre- and post-pubertal RS girls (data not shown).

### Other outcomes including function and pain

Two patients regained the ability to walk towards the end of the 2 years of therapy. Fifteen out of 20 parents reported a decrease in chronic pain and that their daughters started standing physiotherapy. GMFCS level increased significantly (p = 0.020), i.e. the number of patients classified GMFCS level V went from 14 to 7.

**Fig 5:**
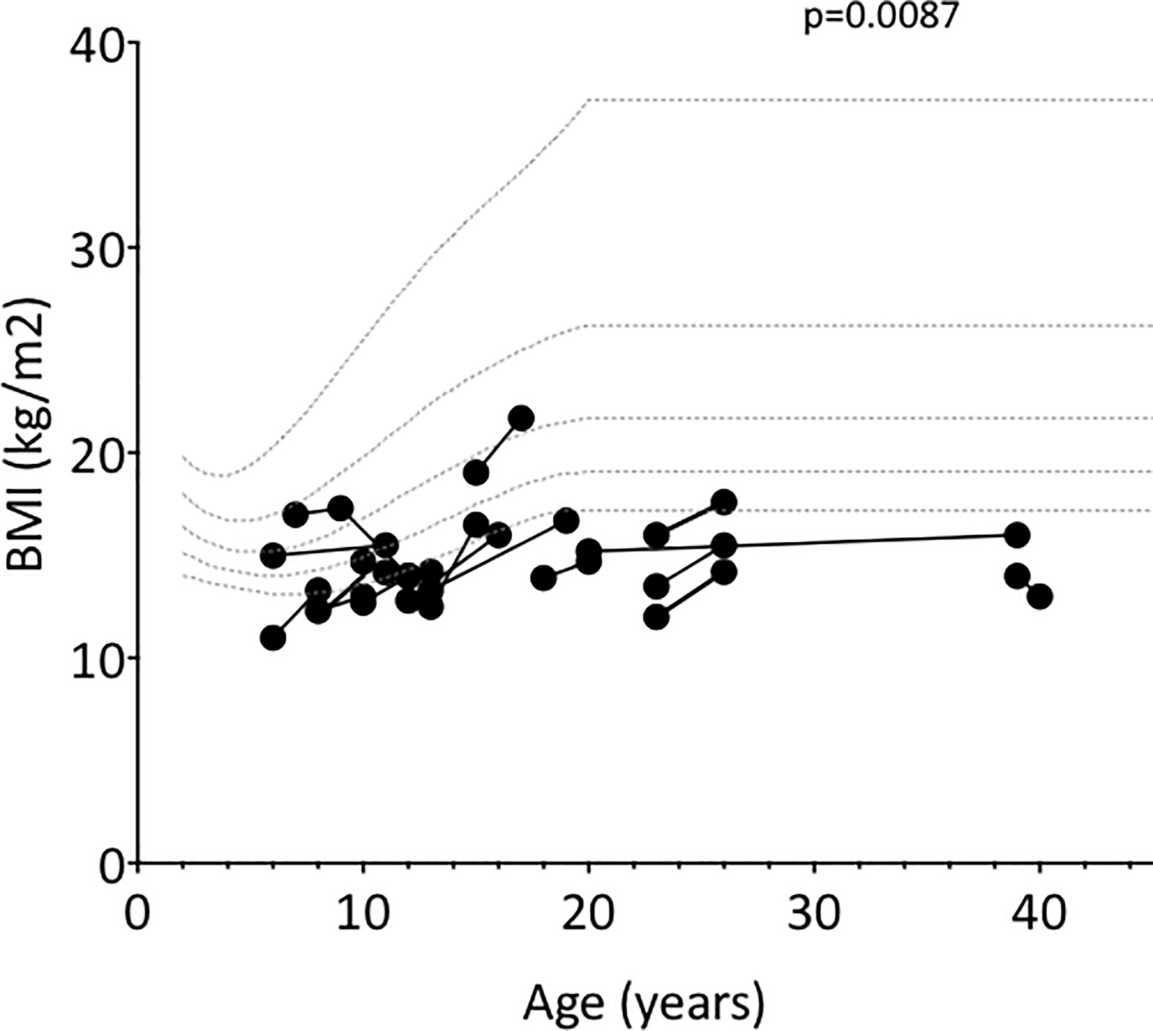
Changes in BMI over the course of treatment.

The median BMI significantly increased between the start and the end of the bisphosphonate therapy, from 13.9 kg/m^2^ [11; 19.04] (z-score -1.5 [-2.4; 0.8]) to 15 kg/m^2^ [13; 21.2] (z-score -1.2 [-1.9; 0.4]), (p = 0.0087 and 0.0306, respectively) (**Fig 5**).

Except for moderate hypocalcemia and fever which occurred mainly at the time of the first infusion, the bisphosphonate infusions (pamidronate in 19 girls and zoledronate in 1 girl) were well tolerated in all patients.

## Discussion

Several reports have already highlighted that patients with RS are at risk of low BMD and fractures [6] [12] [16]. Bone fragility and fractures result in acute and chronic pain and significantly impair the quality of life of disabled individuals. The use of bisphosphonates in children and adults with cerebral palsy (which includes Rett syndrome) is still off-label. On the other hand, the literature suggests that there are sufficient safety and efficacy data to justify their use on compassionate grounds in severe cases of clinical bone fragility (fractures and pain) [19] [20][21] [21][22] [22]. Therefore, since 2009, we have used bisphosphonate treatment in RS patients who present with bone fragility, fractures and/or bone pain.

The patients reported in this manuscript displayed many risk factors for low BMD and bone fragility including a lack of ambulation, insufficient dietary calcium intake, anticonvulsant medication, underweight and delayed puberty or amenorrhea. Most patients had 25OHD levels within the normal range, thereby ruling out vitamin D deficiency as the primary cause of the observed bone fragility. The occurrence of bone fragility in such girls, despite the care given, is in accordance with the data of Jefferson and colleagues showing that genotype, epilepsy, BMI, and mobility affect BMD in girls with RS [12]. We did not show any association between the *MECP2* genotype and the bone mineral density in this cohort of patient (**S1 Fig**). This is similar to findings in patients with cerebral palsy for whom mobility, feeding difficulties, BMI, and the underlying cause of the cerebral palsy itself, have been identified as causes of a low BMD and fractures.

The median BMD measured in our cohort of RS patients was noticeably low associated to a tendency to an increase in bone resorption as shown by the elevated values of urinary calcium excretion, urinary deoxypyridinoline, and serum crosslaps prior to treatment. Conversely, levels of bone formation biomarkers, i.e., alkaline phosphatase and osteocalcin, were on the low side of the normal range. It is worth noting that the patients reported in this manuscript are likely to be different from those described in prospective studies investigating BMD. In fact, the RS of the herein reported series were selected because of fractures and bone pain and may be representative of the most severely affected cases [23].

As observed in other patients affected with cerebral palsy, we observed in this cohort of RS girls upon IV bisphosphonate: a marked increase in BMD, a decrease in calcium to creatinine ratio marker, a reduction in the incidence of fractures, an improvement of the nutritional status (including z-score of BMI) and, more importantly, an improvement of the mobility (GMFCS level). Because our research lacks the rigor of a prospective study, however, we cannot definitively attribute the relative lack of new fractures to the bisphosphonate therapy only. It is likely that the multidisciplinary care including regular nutritional counselling and support, adjustment of daily caloric, calcium intake and 25OHD supplementation, contributed to improve the bone health.

The appropriate duration and dose of the bisphosphonate treatment in patients with disuse bone fragility is still a matter of debate. Based on the literature and on the finding that lumbar spine BMDs are still profoundly low after 1 year on IV bisphosphonates, we designed our program to last arbitrarily for 2 years. Indeed, the median age at the beginning of the anti-resorption therapy was about 12.5 years and, therefore, after 2 years of therapy, we can assume that most of the patients would have completed their pubertal development, and hence that sex steroids would have been able to contribute to the gain or maintenance of the BMD values. We do not know whether we should aim at the normalization or near-normalization of the BMD in these patients, because bisphosphonates are stored for years in the mineralized tissues leading to a prolonged exposure to the therapy, and because an efficacy in reducing fractures and pain is observed even if most patients do not reach the lower end of the normal value of BMD. In fact, we observed a sustained improvement in lumbar spine BMD even after the cessation of the IV administration. It remains of crucial importance to pursue a multidisciplinary approach to limit bone loss as much as possible, with regular clinical and BMD work-ups, and the promotion of good nutrition, calcium and vitamin D intake and weight-bearing [24].

In addition to being retrospective and without a comparison group, a weakness of our study is that we did not assess bone pain, a strong marker of disuse bone fragility. We need to include the quantification of pain in our daily practice through the use of instruments designed for patients with disabling conditions, for example, scales allowing observational assessments such as the Children’s Hospital of Eastern Ontario Pain Scale or the Non-Communicating Children's Pain Checklist (30 items).

Criteria for the use of IV bisphosphonates in these patients are still open to discussion. Children with cerebral palsy -including RS- who have already had a fracture are at high risk of future fractures. Therefore, like others, we consider that IV bisphosphonates should be initiated in patients with RS who have both a low BMD and a history of at least one fracture [22] [25]. Recently, “Clinical Guidelines for Management of Bone Health in Rett Syndrome Based on Expert Consensus and Available Evidence” were published in Australia suggesting that the use of bisphosphonates should be considered in patients with RS with vertebral fractures with or without low BMD z-scores or RS with clinically significant fractures and a low BMD z-score [12].

## Conclusion

Patients with RS are at risk of bone fragility, bone pain and fracture. IV bisphosphonates are easy to use and well tolerated when administered by an experienced team. They improve BMD and likely decrease the incidence of fracture. However, their use should be carefully monitored as long-term data in other settings have revealed unexpected events such as atypical fractures [26]. Both our data and the lack of supporting literature indicates avenues for further research to enhance our understanding of how to prevent fracture and improve bone density in patients with RS. Prospective use of bisphosphonates in the context of monitored clinical trials is needed to unravel the putative efficacy of the drug in this population.

## Supporting information

S1 Fig MECP2 mutations and bone mineral density in 19 children. (TIFF)

S1 Table MECP2 mutations in 19 patients (one patient had a mutation mentionned in the medical notes but the type of mutation was not available). (DOCX)
