# Supplementary figures and images for: Lower incidence of fracture after IV bisphosphonates in girls with Rett syndrome and severe bone fragility
Source: PLoS One. 2017 Oct 26;12(10):e0186941. doi: 10.1371/journal.pone.0186941 (PMC5658100; doi:10.1371/journal.pone.0186941)

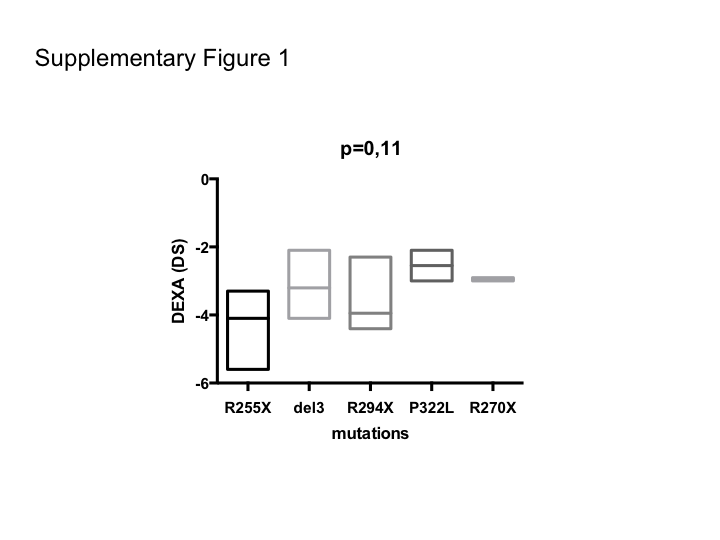

Supplement: S1 Fig — (TIFF) [file pone.0186941.s001.tiff]
