# Supplementary material for: Lower incidence of fracture after IV bisphosphonates in girls with Rett syndrome and severe bone fragility
Source: PLoS One. 2017 Oct 26;12(10):e0186941. doi: 10.1371/journal.pone.0186941 (PMC5658100; doi:10.1371/journal.pone.0186941)
Supplement: S1 Table — (DOCX) [file pone.0186941.s002.docx]

**Supplementary Table 1:** *MECP2* mutations in 19 patients (one patient had a mutation mentionned in the medical notes but the type of mutation was not available)

| Mutations | Patients |
| --- | --- |
| \| R255X \| \| --- \| | 4 |
| \| Del 3 \| \| --- \| | 4 |
| \| R294X \| \| --- \| | 6 |
| \| P322L \|  \| \| --- \| --- \| | 2 |
| R270X | 2 |
| Del 3-4 | 1 |
